# Effect of new type extrusion modification technology on supramolecular structure and *in vitro* glycemic release characteristics of starches with various estimated glycemic indices

**DOI:** 10.3389/fnut.2022.985929

**Published:** 2022-08-15

**Authors:** Bo Li, Yanjun Zhang, Wanru Luo, Jin Liu, Chongxing Huang

**Affiliations:** ^1^College of Light Industry and Food Engineering, Guangxi University, Nanning, China; ^2^Spice and Beverage Research Institute, Chinese Academy of Tropical Agricultural Sciences, Wanning, China; ^3^Key Laboratory of Processing Suitability and Quality Control of the Special Tropical Crops of Hainan Province, Wanning, China; ^4^Women's and Children's Hospital of Wanning, Wanning, China

**Keywords:** glycemic release rate, improved extrusion cooking technology, staple crop starch, fine supramolecular structure, principal component analysis

## Abstract

Nowadays, the highly effective modified technology to starch with various digestibility is gaining interest in food science. Here, the interactions between glycemic release characteristics and fine supramolecular structure of cassava (ECS), potato (EPS), jackfruit seed (EJFSS), maize (EMS), wheat (EWS), and rice starches (ERS) prepared with improved extrusion modification technology (IEMS) were investigated. The crystalline structures of all extruded cooking starches changed from the A-type to V-type. IEMS-treated cassava, potato, and rice starches had broken α-1.6-glycosidic amylopectin (long chains). The others sheared α-1.4-glycosidic amylopectin. The molecular weight, medium and long chain counts, and relative crystallinity decreased, whereas the number of amylopectin short chains increased. The glycemic index (GI) and digestive speed rate constant (*k*) of ECS, EPS, EJFSS, and EWS were improved compared to those of raw starch. Although EMS and ERS had degraded molecular structures, their particle morphology changed from looser polyhedral to more compact with less enzymolysis channels due to the rearrangement of side chain clusters of amylopectin, leading to enzyme resistance. The starch characteristics of IEMS-treated samples significantly differed. EPS had the highest amylose content, medium chains, long chains, and molecular weight but lowest GI, relative crystallinity, and *k*. ERS showed the opposite results. Thus, IEMS may affect starches with different GIs to varying degrees. In this investigation, we provide a basis for wider applications of conventional crop starch in the food industry corresponding to different nutrition audience.

##  Highlights

- Various glycemic indices (GI) starches were treated by new extrusion cooking (IEMT).
- GI of IECT rice, maize starch was lower than the native, that differed with others.
- Crystal structure of IECT starches became V-type from A-type after IECT treatment.
- The degraded mode of amylose glycosidic bond was associated with amylose content.
- IECT starches showed lower molecular weight, fraction of long chain than the native.

##  Introduction

Starch is a major macronutrient required by humans and is frequently extracted from unripe fruit pulp, seeds, roots, tubers, stems, and grains of crops such as jackfruit, cassava, rice, wheat, potato, and maize. Because different starch sources have various molecular encapsulation reaction of glucan chains of amylopectin and amylose, starches are defined as C_A/B_-types, A-types, and B-types based on their crystal structure (1). The Va, Vh and B+V-type crystal structure is usually characteristic of modified starch (2). Starches from different crops have unique supramolecular structures causing such foods to have variable digestibility (3). According to Li et al. (4), jackfruit seed and cassava starches are medium-level blood glucose foods, potato starch is a low-level blood glucose food, and starches of common staple crops such as rice starch are high-level blood glucose foods. This probably produced distinct modification mechanisms between them. However, most native starches are difficult to digest in the initial digestion stage because of their low rate of enzymatic hydrolysis (*k*), preventing timely nutrient release from native starches in the human body. Furthermore, native starches have a high weight-average molar mass (Mw), high amylopectin long-chain distribution, and high relative crystallinity (Rc), leading to a slow glycemic release. And it is difficult to maintain the necessary nutrition of the human body (2). Therefore, native starches should be modified to improve their digestion rate in the human intestine. The various glycemic release characteristic of cassava, potato, jackfruit seed, maize, wheat, and rice starches were due to the significantly different amylose content, crystalline structures, chain length distribution, molecule weight and particle morphology. This probably also might produce the distinct modification mechanisms between them (1, 4). In addition, based on Zhang et al. (2), extrusion modification could be used to prepare a pregelatinized starch. This kind of starch could use directly as an edible food to provide essential nutrients.

The supramolecular structures and digestibility characteristics of native starches can be changed during extrusion cooking, high-pressure microfluidization, hydrothermal treatment, and annealing treatment (5, 6). Extrusion cooking have been becoming a common modification technology, which was a continuously elevated temperature process with a fast-heating rate. During extrusion cooking process, the moistened expandable starch is physically swelled through an extremely high shear stress, temperature and pressure compared with those of other modification methods. Moreover, the improved extrusion modification technology was initially mentioned by Zhang et al. (7). It can alter the molecular structure of starch by using more mild extrusion cooking conditions (lower shear stress and temperature) and higher intensity of pressure than those of a common extrude. We previously developed an improved extrusion modification (IEMS) system as a gel-modification pattern for changing starch digestibility. This method can alter the molecular structure of starch because it uses a lower shear stress and temperature and higher pressure compared to that of many other approaches (1). According to Al-Rabadi et al. and Zhang et al., as the starch is sourced from diverse plant varieties, the changes of supramolecular structure, *in vitro* glycemic release rate, and estimated glycemic level of starch samples could vary greatly after treated by extrusion cooking. In our previous study, we used IEMS to explore the digestion mechanism of the resistant starch content in JFSS (5). However, the supramolecular structures and *in vitro* digestive kinetics of extruded cooking staple crop starch with various estimated glycemic indices have not been compared, limiting the exploitation and utilization of starch resources from staple crops. Based on the various digestibilities of extrusion cooking starch with various estimated glycemic indices, starch can be produced as an excellent base for thickeners, stabilizers, and potential wall materials for microencapsulation in starch-basis processing industries., to improve the rate of nutrient release from food.

Therefore, jackfruit seed (JFSS) and cassava, rice, wheat, potato, and maize starches were extracted and used to measure the molecular weight distribution, branching chain distribution of amylopectin, crystallization characteristics, amylose content, digestive kinetics, and estimated glycemic index after IEMS treatment. The possibly distinct *in vitro* digestive mechanism of starch with various glycemic indices prepared by IEMS was further analyzed. This study provides a basis for comprehensive application of starches of various digestibility levels in the food industry.

## Materials and methods

### Materials

Fruits of the Malaysia 1 jackfruit cultivar were collected in 2019 from the Xinglong Tropical Botanical plantation (Wanning, Hainan, China) and assigned voucher number 202009. Cassava, maize, potato, wheat, and rice were purchased from a local market (Nanning, Guangxi, China).

### Preparation of different starches

Jackfruit seeds, cassava, maize, potato, wheat, and rice were pre-treated quickly after drying in a drying cabinet at 50°C. The pre-treated samples were milled with distilled water (1:3 w/w) for 2 min in a colloid grinder. Then pH of starch slurry was adjusted to 7.0, mixed with neutral protease solution (0.015% w/w) (Alphalase NP, 240,000 U/g, Sigma, St. Louis, MO, USA), and transferred into a constant-temperature shaking bed (60°C, 9 h and 150 rpm). The mixed solution was filtered through a filter cloth (200 mesh) and centrifuged (3,000 × *g*, 5 min) to remove residual brown impurities. After repeated washing with distilled water, the sediment was collected. The centrifuged sediments were repeatedly cleaned and collected. The resulting wet starch was dried under vacuum (50°C) until the moisture content was lower than 13 g/100 g. After passing through a 200-mesh sieve, the dry starch was stored in a vacuum dryer until use (8).

### Modification of different starches with IEMS

Extrusion modification experiments were conducted in a twin-screw extruder equipped with a barrel with self-adapting multiple-region temperature system (7). The length-width ratio of the extruder screw was approximately 19.5:1, and the diameter was 100 mm. The self-adapting multiple-region temperatures were adjusted to 50, 65, 85, 95, and 100°C, respectively. The starch samples with 30% w/v water prepared by that calculated water and the raw starch with certain quality (dry basis) were added to a flour mixing machine within the extrusion equipment (13 rpm, 5 min). The screw speed range was 25 rpm. The starch was filled into a revolving feed system at 13 rpm. The extrudates were cut with a rotary cutter at 6 rpm, dried under vacuum, and stored in a vacuum dryer. The extrusion-cooked JFSS, cassava, maize, potato, wheat, and rice starches were named as EJFSS, ECS, EMS, EPS, EWS, and ERS, respectively.

### *In vitro* digestibility of extrusion-cooked starches of various estimated glycemic levels

The proportions of resistant starch (RS), slowly digestible starch (SDS), and rapidly digestible starch (RDS) were determined as described by Englyst et al. (9) with slight modifications. The mixed enzyme solution, which contained ≥225 U/mL of amyl-glucosidase and 20 U/mL of porcine pancreas α-amylase (Megazyme, Wicklow, Ireland), was transferred to 20 mL sodium acetate buffer (0.1 M, pH 5.2) with 1 g of starch. The mixed liquids were incubated in a constant temperature water bath (36.5–37°C, 180 rpm). The enzymes were inactivated by adding 70% ethanol (20 mL) to the supernatant (0.5 mL) at 20- and 120-min intervals. This solution was centrifuged (4,000 × *g*, 10 min), and the supernatant was collected to determine the glucose content using a glucose oxidase-peroxidase method (Megazyme) and a spectrophotometer (UV-2700, Shimadzu, Kyoto, Japan) at 510 nm. The RDS, SDS and RS contents in extrusion-cooked starches were calculated as follows:


$$\begin{array}{lll} RDS\left(\%\right) & = & \frac{\left(G_{20}-G_{F}\right)\times 0.9}{TS}\\ SDS\left(\%\right) & = & \frac{\left(G_{120}-G_{20}\right)\times 0.9}{TS}\\ RS\left(\%\right) & = & \frac{\left[TS-\left(RDS+SDS\right)\right]}{TS} \end{array}$$

where G_20_ (%) is the released content of glucose within 20 min, G_120_ (%) is the released content of glucose at 120 min, G_F_ (%) is the free glucose content, and TS (%) is the total starch content.

### Characteristics of *in vitro* digestibility kinetics of starch

The *in vitro* digestibility kinetics of starch was determined as described by Goñi et al. (10) with slight modifications. 15-mL sodium acetate buffer solution was mixed with 200 mg starch (dry basis). Then Amyloglucosidase (15 U/mL) and porcine pancreatic α-amylase (290 U/mL) (total 10 mL) (Megazyme) and seven glass were added to that solution. The mixed liquids were reacted in a shaking bed (37°C, 150 rpm). At 10-, 20-, 30-, 60-, 90-, 120- and 180-min, absolute ethanol (4 mL) was added to the supernatant (0.5 mL). This solution was centrifuged at 6,000 × *g* for 15 min at 10°C, and the supernatant was transferred to glucose oxidase-peroxidase to analyze the glucose content. The percentage of enzymatic hydrolysis was calculated as follows (11):


$$\begin{array}{l} Percentage\;of\;hydrolyzed\;starch\;\left(\%\right)=\frac{G_{t}\times 25\times 0.9}{200}\times 100 \end{array}$$

where 0.9 is the transformation coefficient from starch to glucose (162/180 w/w), 25 is the dilution factor, and glucose concentration within t min was defined as G_t_.

The equilibrium concentration (*C*_∞_) and speed rate constant (*k*) (*h*^−1^) were obtained from the enzyme hydrolysis curves, and the first-order formulas were as follows:


$$\begin{array}{lll} C & = & C_{\infty }\left(1-e^{-kt}\right),C_{\infty }\leq 100\%\\ AUC & = & C_{\infty }\left(t_{f}-t_{o}\right)-\left(\frac{C_{\infty }}{k}\right)\left[1-exp-k\left(t_{f}-t_{0}\right)\right] \end{array}$$

where AUC is the area under the fitted curve, t_0_ and t_f_ are the initial and final times of hydrolysis, and *t* is the time of *in vitro* digestibility kinetics (min).

### Predictive glycemic index of starch

The area of the *in vitro* digestibility kinetics curve was calculated as the hydrolysis index (HI) and estimated glycemic index (GI) corresponding to white bread as a reference using the following equation (12):


$$\begin{array}{lll} HI & = & \frac{AUC\;\left(sample\right)}{AUC\;\left(white\;bread\right)}\\ GI & = & 39.71+\left(0.549HI\right) \end{array}$$

### Granule morphology analysis

As described by Zhang et al. (8), a scanning electron microscope was used to observe the granule morphology of starch samples (Quanta-200, FEI Company, Hillsboro, OR, USA). The accelerating voltage was set to 15 kV and magnification was 60 × and 500 ×.

### Fine structure of starch samples

According to Bi et al. (13), the fine structure was generally analyzed by determining the debranched chain length distributions. Pullulanase (10 μL; 1,000 NPUN/g, 50 mM, pH 6, Sigma-Aldrich) was mixed with 40 mg starch for debranching. Short chains [A chains, degree of polymerization (DP): 6–12], middle short chains (B1 chains, DP: 13–24), middle long chains (B2 chains, DP 25–36) and long chains (B3+ chains, DP: ≥ 37) of starch samples were analyzed in a Cabopac PA200 column (3 × 250 mm, Dionex Corporation, Sunnyvale, CA, USA) using high-performance anion-exchange chromatography with pulsed amperometric detection (ICS-5000, Dionex Corporation) connected to a ED50 electrochemical detector.

### Molecular structure analysis

As described by Zhang et al. (14), the molecular structure was analyzed by determining the molecular weight distribution. Completely dissolved solution (starch samples/dimethyl sulfoxide was 2 mg/mL, 90°C, 24 h) was evaluated with an absolute molecular weight analysis system including multi-angle laser light-scattering detector (Wyatt Technology Corporation, Santa Barbara, CA, USA), refractive index detector (Wyatt Technologies), and high-performance size-exclusion chromatography (Wyatt Technology Corporation). The guard column, Shodex OHpak SB-804 HQ and Shodex OHpak SB-806 HQ (Showa Denko K.K., Tokyo, Japan) Phenogel columns were used. The column temperature was 60°C, and flow rate of the dimethyl sulfoxide mobile phase was 0.3 mL/min. The sample injection consisted of 100 μL. Data obtained using this system were analyzed with Astra software (version 5.3.4, Wyatt Technology).

### Crystal structure and degree of gelatinization analysis

For crystal structure analysis, an X-ray diffractometer (Bede XRD Di System, Durham, UK) operated at 40 kV, 4 to 40°, 200 mA, and 0.154 nm CuKα radiation was used. Relative crystallinity (Rc) was calculated using MDI Jade v6.5 (15). It was measured by that the ratio of peak cell area to total area.

The degree of gelatinization (DG) was defined as the glucose content of per gram of gelatinized starch sample after enzymolysis. Therefore, DG could be carried out based on method of enzymatic detection. 100 mg starch sample were passed 200 mesh sieve and hydrolyzed by amyloglucosidase (50 U, 37°C, 30 min). The glucose oxidase-peroxidase method used to detect glucose content. The DG was measured by the ratio of starch samples to fully gelatinized starch standard.

### Proportions of amylose and amylopectin

According to Chen et al. (1), a mixed liquor composed of 1 mL absolute alcohol and 9 mL of 1 M sodium hydroxide was prepared, which was mixed with 100 mg samples (dry basis) and boiled in a water bath for 15 min. This solution was diluted to 100 mL using distilled water. Next, 2.5 mL of the diluent were further diluted to 50 mL using distilled water. The 0.50 mL of acetic acid solution (1 M) and 1 mL of iodide and iodine (0.0025 M I_2_, and 0.0065 M KI) was used to react with that diluent, then stewing 20 min for Color reaction. The absorbance was measured in an ultraviolet spectrophotometer at 620 nm (SPECORD 250 plus, Analytik Jena, Germany). The standard curve of potato amylose (Alphalase NP, Sigma) was used to calculate the amylose and amylopectin contents.


$$\begin{array}{l} Amylopectin\;\left(\%\right)=\left(1-amylose\left(\%\right)\right)\times 100 \end{array}$$

### Statistical analysis

The means, standard deviations, and principal component analysis (PCA) performed using SPSS (version 20.0; SPSS, Inc., Chicago, IL, USA) were used to determine the interaction between the glycemic release rate characteristics and fine supramolecular structure. The data was analyzed by one-way ANOVA at 5% level of significance. The significance of differences between parameters (at *p* < 0.05) was determined using Duncan's multiple.

## Results and discussion

### *In vitro* nutrition fractions

The *in vitro* nutrition fragments (RDS, SDS, and RS) of EJFSS, ECS, EPS, ERS, EWS, and EMS were shown in Table 1. The RDS, SDS, and RS showed obvious diversities among the six sample types (*p* < 0.05). EPS showed the maximum proportion of RS but minimum RDS values. ERS showed the maximum SDS and lowest RS. The highest RDS content was observed in EWS and lowest SDS content was observed in ECS. Therefore, EPS showed the strongest enzyme resistance, followed by EJFSS, ECS, EMS, and EWS. ERS was also susceptible to enzymolysis. Moreover, according to Zhang et al. (3), cassava, maize, potato, wheat, and rice starches have an A-type crystalline structure, whereas potato starch has a B-type crystalline structure. Wang et al. (16) also found that cooked some mung bean starch and sago starch with B-type crystalline structure also showed significantly gelatinization characteristics at a molecular level, compared with A-type crystalline corn, oats and barley starch. This phenominon might be exlpained by that the diversities of ishort chain aggregates, isolated single helices rregularly and packed structures between B-type crystalline starch and A-type crystalline satrch (16, 17). Therefore, the highest RS in EPS may be explained by that although extrusion cooking starch might all show V-type crystal structure, the different crystal types between native potato starch and other raw samples still have different modification mechanisms, leading to the various relative crystallinity and repetition distance of semicrystalline lamellar. According to Ma et al. (17), for the extruded A-type crystalline starch samples, different nutrition fragments were produced, possibly because of the responsiveness of α-amylase to the hyperfine structure of starch pellets. Our results were similar to those of hull-less barley starch, which showed an RS content of 17–56% after extrusion cooking (18). The RDS content of the extrusion-cooked starch samples (28.32–55.22%) was consistent with that of extruded high-amylose maize flour (RDS 19.32–66.83%); however, the SDS content (30.75–47.66%) was broadly higher than that of extruded high-amylose maize flour (SDS 2.70–36.51%) (19). These differences may be ascribed to the higher degree of amylopectin polymerization in our samples compared to that in extruded high-amylose maize flour.

**Table 1:** *In vitro* nutritionally starch fractions, kinetic equation characteristics of enzymatic hydrolysis and glycemic index of test material.

**Starch samples**	**RDS (%)**	**SDS (%)**	**RS (%)**	**C_∞_(%)**	***k* (h^−1^)**	**HI**	**GI**
EJFSS	46.28 ± 6.41^de^	34.60 ± 3.86^de^	19.11 ± 2.56^b^	82.52 ± 1.44^e^	1.88 ± 0.10^bc^	96.50 ± 1.70^e^	92.69 ± 0.93^e^
ECS	53.99 ± 1.74^ab^	30.75 ± 1.33^f^	15.26 ± 0.40^c^	85.28 ± 3.49^d^	1.61 ± 0.20^d^	99.69 ± 4.11^d^	94.43 ± 2.26^d^
EPS	28.32 ± 0.80^f^	42.13 ± 4.25^b^	29.55 ± 5.05^a^	79.23 ± 1.69^f^	1.43 ± 0.09^f^	92.22 ± 1.62^f^	90.34 ± 0.89^f^
EMS	50.03 ± 5.18^cd^	36.83 ± 1.28^d^	13.13 ± 3.90^cd^	90.42 ± 2.51^b^	1.55 ± 0.13^de^	105.69 ± 2.96^bc^	97.73 ± 1.62^bc^
EWS	55.22 ± 1.79^a^	41.80 ± 1.11^bc^	2.98 ± 0.27^e^	92.25 ± 2.02^a^	1.90 ± 0.13^b^	107.88 ± 2.38^a^	98.93 ± 1.30^a^
ERS	50.44 ± 0.52^c^	47.66 ± 0.75^a^	1.90 ± 1.26^f^	90.78 ± 2.45^b^	2.06 ± 0.18^a^	105.17 ± 2.88^ab^	98.00 ± 1.58^ab^

*RDS, SDS and RS were rapidly digestible starch, slowly digestible starch, and resistant starch. C_∞_ and k represented equilibrium concentration and first-order kinetics constant. HI and GI were predictive hydrolysis index and glycaemic index. Samples with different letters in the same column are significantly different at P < 0.05*.

The RDS contents of all extruded cooking samples were notably higher compared to those in the corresponding raw samples, whereas the RS content was much lower (*p* < 0.05) (Table 1, Supplementary Table S2). After IEMT treatment, the SDS contents of the JFSS and potato starch were significantly higher, while those of the other native starches were significantly lower (*P* < 0.05). These results indicate that most RS and SDS in ECS, ERS, EWS, and EMS were converted into RDS, whereas the RS in EJFSS and EPS was converted to both RDS and SDS. According to Zhang et al. (19), extrusion promotes the formation of defective crystalline regions and disordered semi-crystalline lamellae. This may lead to improved digestibility of starch samples after extrusion cooking. In addition, based on Li et al. (4), the different conversion abilities of nutrition fragments between JFSS and potato starch compared to other kinds of starch, may be related to the higher amylose content in JFSS (27.01%) and potato starch (24.82%) than in the other starch samples (3.25–21.65%). This led to higher formation of nearly perfect crystals and better short-range order structures for JFSS and potato starch, resulting in different levels of digestibility after extrusion. Changes in the *in vitro* nutrition fraction contents of the six types of starch during extrusion cooking were similar to those of extruded waxy rice flour, in which the RDS increased from 50.50% to 78.88%, RS decreased from 14.74 to 2.36%, and SDS decreased from 34.77 to 6.84% (20).

However, the *in vitro* nutrition fractions contents suggested by Englyst et al. (9) were not confirmed in accurate enzymolysis studies (21). Hence, the *in vitro* glycemic release rate and estimated glycemic level should be analyzed to assess the digestibility of native starch extrudates.

### *In vitro* digestive kinetics of IEMS starches

**Figure 1:**
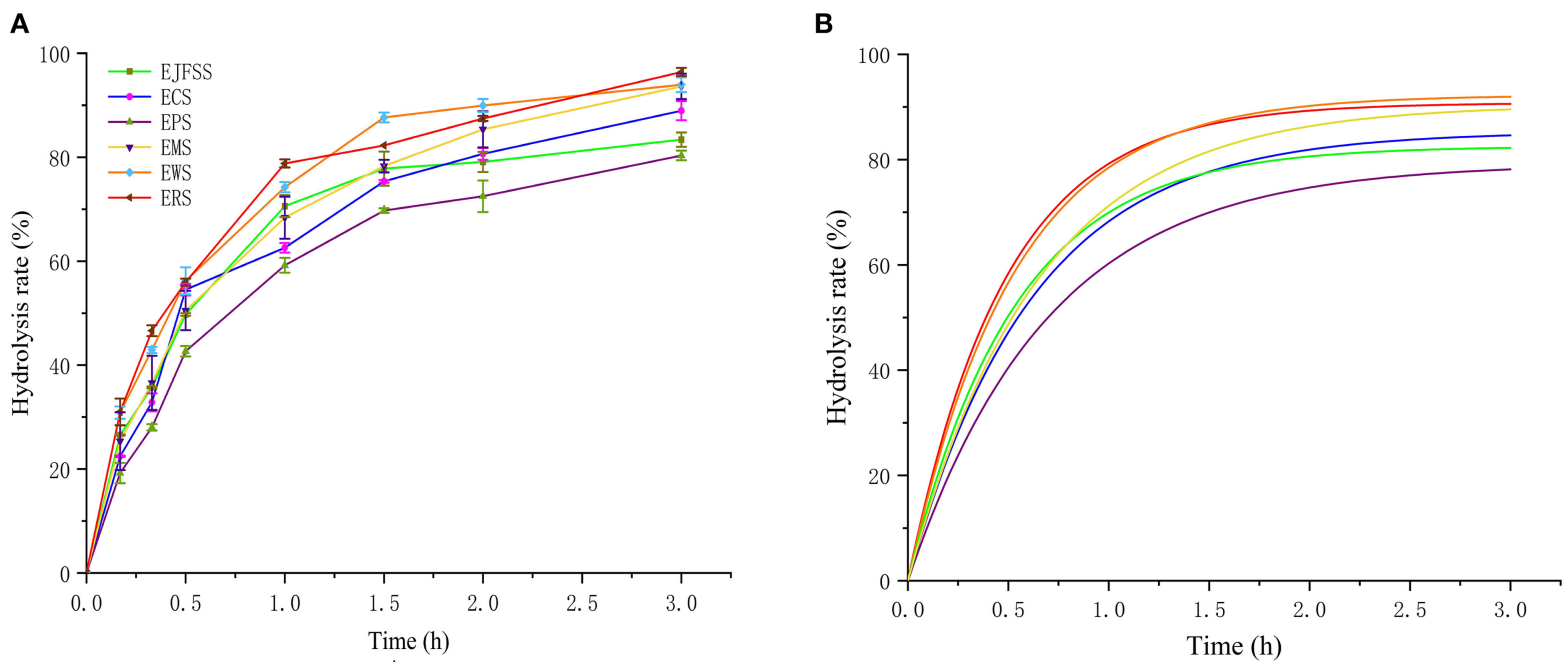
Primitive curve **(A)** and forecast curve **(B)** of *in vitro* hydrolysis of different extrusion-cooked starch samples.

As shown in the primary and fitted digestive curves in Figures 1A,B, the enzymatic hydrolysis rates followed the order: ERS > EWS > EMS > ECS > EJFSS > EPS. Le Corre et al. (22) reported that starch digestion mainly occurs in the amorphous region formed by amylose in the entangled state and branch points of low DP amylopectin. Therefore, the various extrudate digestion rates may be explained by their distinct distribution patterns of entangled state amylose, which forms a differently compacted amorphous structure. The hydrolysis curve of extrudates showed a rapid increase from 0–75 min, after which the hydrolysis curve increased slowly to a maximum over 75–180 min (Figures 1A,B, Supplementary Table S1). Zhang et al. (8) also reported that extrusion-cooked JFSS undergoes faster hydrolysis in 0–60 min compared to that in 90–180 min. The digestion rates of the starch extrudates were 59.21–78.80%, 72.49–89.95%, and 80.33–96.39% at 60, 120, and 180 min, respectively. This result indicates that all extrudates were weakly resistant to digestion. Furthermore, the hydrolysis curves of extrudates samples were notably higher compared to those of the corresponding native samples (*p* < 0.05) (Figures 1A,B, Supplementary Tables S1, S2). This occurred possibly because the fragments of amylose and amylopectin reassociated, and the polymers were reconstituted with weak intermolecular forces during the retrogradation stage of extrusion cooking starch based on Zhang et al. (19, 20). Zhang et al. (8) reported similar results; at all time intervals, the hydrolysis ratio of extrusion-cooked JFSS (0–91.68%) exceeded the corresponding hydrolysis ratio of native starch (0–31.84%).

The C_∞_ values were in the following order: EWS > ERS ≈ EMS > ECS > EJFSS > EPS (Figure 1B, Table 1). The starch sample extrudates had significantly increased C_∞_ values compared with those of the native starches (Table 1 and S1 *p* < 0.05), whereas ERS and EMS showed the opposite trend. The C_∞_ values of ERS (90.78%) and EMS (90.42%) were considerably lower than those of raw rice starch (91.06%) and maize starch (92.05%), respectively; these results differed from those of enzymatic hydrolysis curve analysis. C_∞_ is significantly correlated with the enzymatic hydrolysis time intervals (12) and *k* (5). C_∞_ represents the predicted final glycemic release content after the digestive reaction. The difference may be attributed to the various *k* values among EJFSS, ECS, EPS, ERS, EWS, and EMS, resulting in different C_∞_ values. To determine the mechanism of the change in C_∞_ during extrusion cooking and explain the differences among the results, the *k* of native starch extrudates was analyzed.

ERS and EWS had higher *k* values than EJFSS, ECS, EPS, and EMS, indicating faster glycemic release in ERS and EWS and leading to higher C_∞_ than that in the other extrudates. This may have led to a notably lower C_∞_ of ERS and EWS compared to those of raw starches, whereas the others were higher (Table 1, Supplementary Table S2). Based on previous studies (17, 23), the branching chain distribution properties of amylopectin and crystallization characteristics may have caused differences in *k* among the extrudate samples. Furthermore, *k* of extrusion cooked starches (1.55–2.06 *h*^−1^) exceeded that of raw starches (Table 1, Supplementary Table S1). This may be because the enzymatic hydrolysis site transforms from the amorphous region near the particle surface into the amorphous structure near the center in starch particles, as reported by Jiang et al. (24). AlRabadi et al. (25) reported similar results, where the *k* value of sorghum starch extrudates (2.12 *h*^−1^) was higher than that of raw starch (0.20 *h*^−1^).

### Glycemic index analysis

According to Goñi and Valentín-Gamazo (11), a high blood glucose level was considered as GI ≥ 70. A GI value between 55 and 69 is considered as a medium glycemic index level and <55 indicates a low glycemic index level (10). The HI and GI values are shown in Table 1. All extrusion cooking samples had high blood glucose levels (GI: 90.34–98.93, HI: 92.22–107.88). The HI and GI values were in the following order: EWS > ERS ≈ EMS > ECS > EJFSS > EPS, possibly because of the various quantities of more ordered mass fractal structure of the extrudates (1). The HI and GI values of the extrusion-modified samples remarkably surpassed those of raw starches (*p* < 0.05), except for ERS and EMS (Table 1, Supplementary Table S2), possibly because the numerous branch linkages in the crystallites caused a higher *k* but a lower C_∞_ in ERS and EMS than in the other samples, as reported by Li et al. (5). The GI of amaranth starch extrudates showed a similar result; the GI value (91.2) of extrusion cooked starches significantly exceed that of native starch (87.2) (26).

### Granule morphology

**Figure 2:**
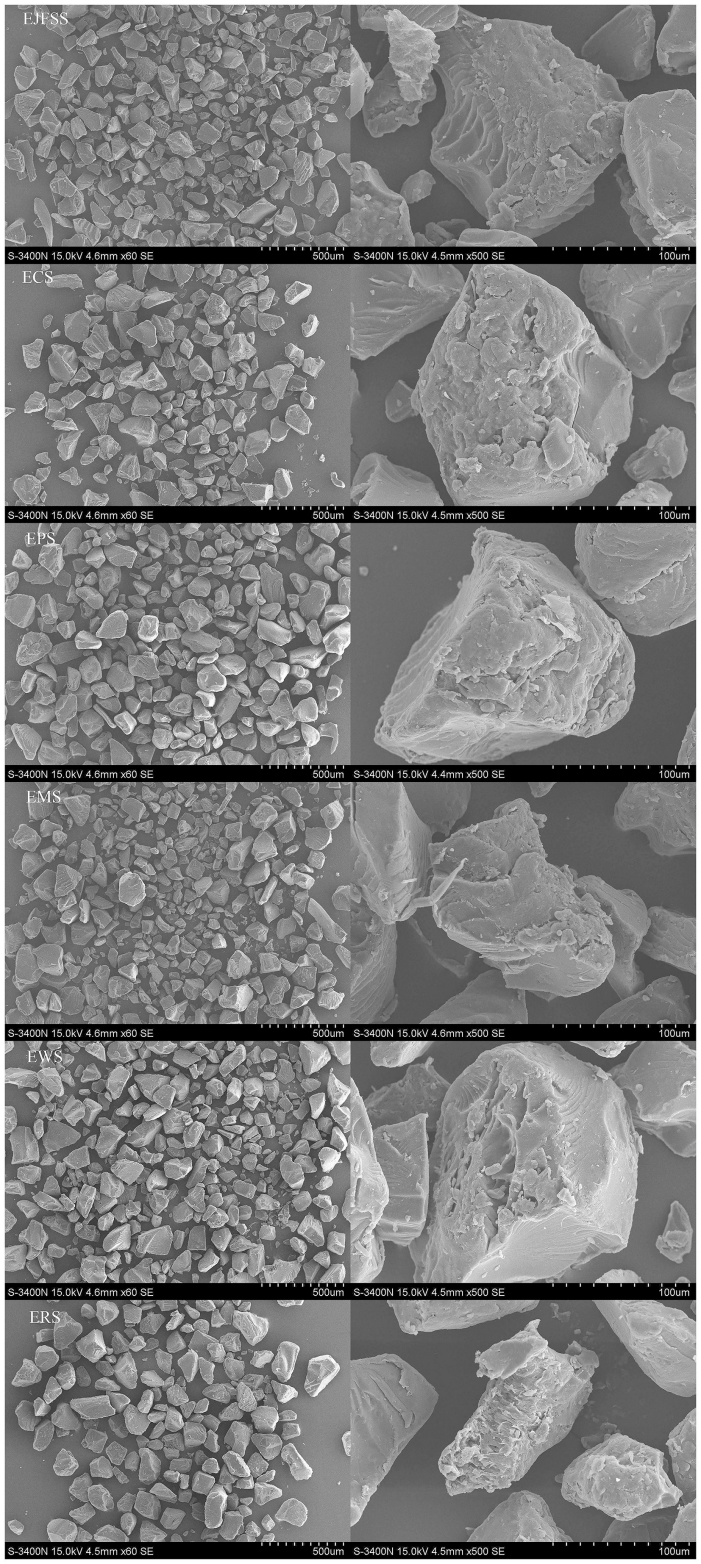
Micrographs of different types of starch extrudates at 60 × (left) and 500 × (right) magnification.

SEM and the supramolecular structure can be used to investigate the *in vitro* glycemic release mechanism of particles after IEMS treatment. Therefore, we examined the granule morphology of starch extrudates. All extrudate samples showed irregular shape (Figure 2). The EPS granules had more compact surfaces with fewer pits compared to those of EJFSS, ECS, ERS, EWS, and EMS granules. EWS had the loosest and most void-distributed granule surfaces. EWS molecules may break more easily during the hydrolysis of amyl-glucosidase and α-amylase than the other samples, whereas EPS showed significant opposite trend (27). Similarly, Faraj et al. (18) showed that the degree of damage to the extrudate granule morphology of different types of barley flour significantly differed. Moreover, the EJFSS, ECS, EPS, and EWS granule morphology exhibited numerous large emulsion bumps with concave holes (Figure 2), in contrast to the smoother, smaller, and round or bell-shaped native starch granule surfaces observed previously (4). Thus, the extrudate granules may have been seriously damaged after IEMS treatment, leading to superior digestibility compared to that of their raw starches (28). The ERS and EMS granules had concave holes and large sizes, whereas their native starches had abundant small pores randomly distributed on the rough surface. Therefore, ERS and EMS had a poor GI value compared with that of their raw starches. Román et al. (29) also found a high degree of granule morphology disruption in extruded maize flour.

### Supramolecular structure

#### Absolute molecular weight distribution of starch samples

**Figure 3:**
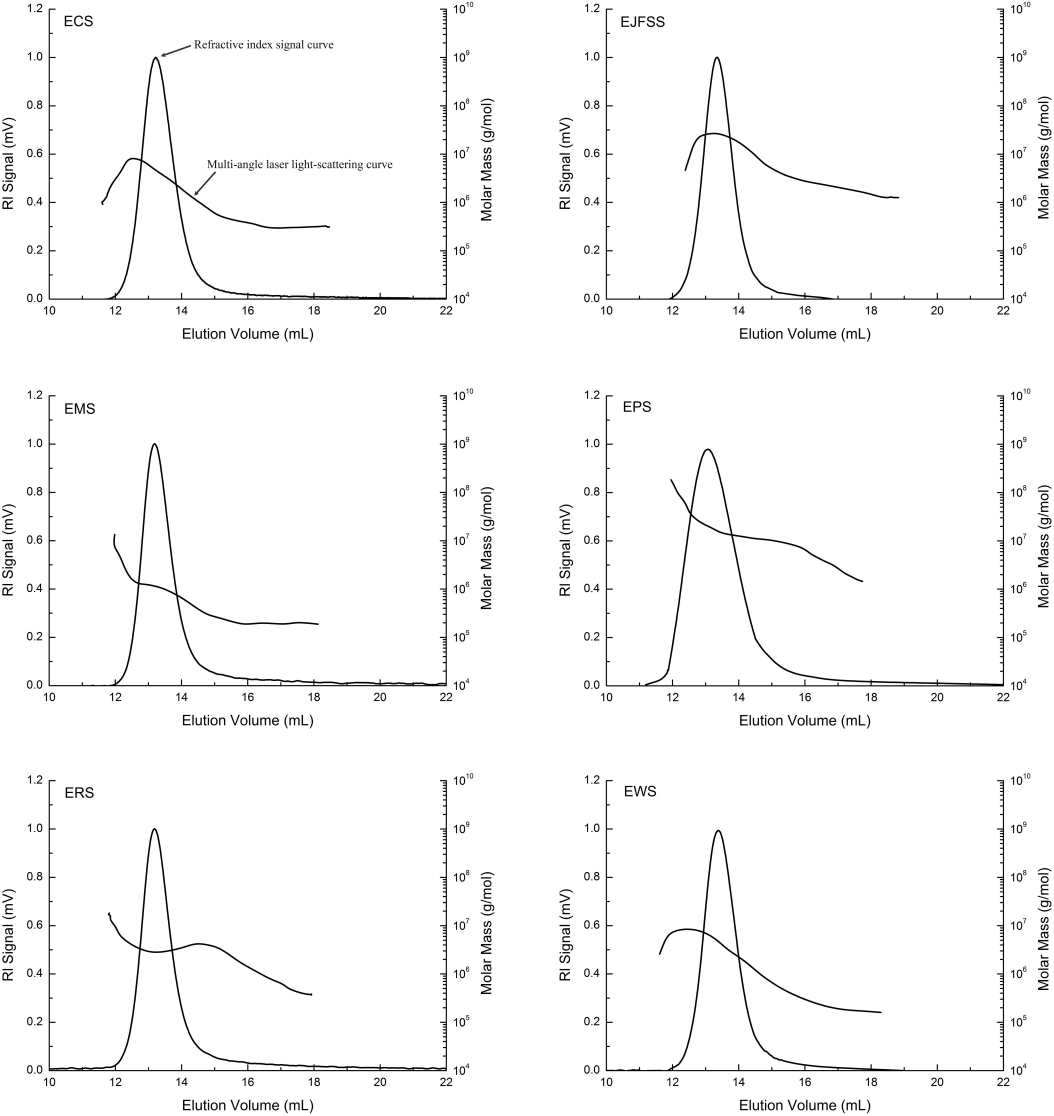
Molecular weight and distribution curve of starch samples.

The absolute molecular weight parameters of EJFSS, ECS, EPS, ERS, EWS, and EMS were 0.30–1.31 × 10^7^ g/mol (Mw), 0.26–1.13 × 10^7^ g/mol (Mn), 53.9–125.4 nm (Rg), and 1.06–2.24 (PI), respectively, and all absolute molecular weight parameters of EPS, EJSS, and ECS were higher than those of EMS, EWS, and ERS (Table 2, Figure 3). This indicates that EMS, EWS, and ERS had looser molecular structures, a weaker force between molecules, and lower molecular weight dispersion compared to those of EPS, EJSS, and ECS (30). The higher Rg, PI, and average molar mass of EPS, EJSS, and ECS may be associated with their relatively lower digestion rates than those of EMS, EWS, and ERS. In addition, Mw chromatogram of starch samples in this study only displayed a single-peak elution curve, that differed with the report of Liu et al. (31) who found the doublet-peak elution curve including amylose and amylopectin curve. This difference might be attributed to the diversity of sensitivity of the MALLS-RI system, the different analysis conditions or variations in the data evaluation techniques.A similar study reported that the Mw of extrusion-cooked waxy maize starch was 40–336 × 10^6^ g/mol (32).

**Table 2:** The fine supramolecular structures of different types of extrusion modification starches.

**Starch sample**	**DP** **6-12**	**DP** **13-24**	**DP** **25-36**	**DP ≥** **37**	**Mn (×10^7^)**	**Mw** **(×10^7^)**	**Rag** **(nm)**	**PI**	**Amylose** **(%)**	**Amylopectin** **(%)**	**Ratio** **(%)**	**Rc** **(%)**
EJFSS	35.47^b^	44.91^d^	12.67^cde^	6.95^b^	0.36^c^	0.81^b^	73.7^c^	2.24^a^	26.98 ± 1.50^b^	73.02 ± 1.50^e^	36.98 ± 2.81^b^	16.22^a^
ECS	32.66^de^	47.09^b^	13.48^bc^	6.77^bc^	0.61^b^	0.79^bc^	68.3^d^	1.29^b^	24.08 ± 0.94^cd^	75.93 ± 0.94^d^	31.72 ± 1.63^cd^	15.10^b^
EPS	32.06^ef^	44.67^de^	15.81^a^	7.46^a^	1.13^a^	1.31^a^	125.4^a^	1.21^bcd^	31.34 ± 0.40^a^	68.66 ± 0.40^f^	45.65 ± 0. 83^a^	9.28^f^
EMS	34.49^bc^	48.61^a^	12.67^cde^	4.23^e^	0.35^cd^	0.43^d^	62.3^e^	1.17^de^	20.56 ± 0.25^e^	79.44 ± 0.25^b^	25.87 ± 0.39^e^	12.29^cde^
EWS	43.09^a^	44.37^ef^	10.00^f^	2.54^f^	0.26^ef^	0.30^f^	53.9^f^	1.20^bc^	22.50 ± 1.20^d^	77.50 ± 1.20^c^	29.05 ± 2.00^d^	12.62^cd^
ERS	33.65^cd^	46.01^c^	14.08^b^	6.26^cd^	0.33^cde^	0.35^de^	115.7^b^	1.06^f^	9.63 ± 1.40^f^	90.37 ± 1.40^a^	10.67 ± 1.71^f^	13.92^c^

*DP represented the chains degree of polymerization. Mn and Mw were weight-average and number-average molar mass molar mass. Rg and PI were radius of gyration and Mw/ Mn. Rc represented relative crystallinity of starch molecular. Ratio was Amylopectin content/Amylose content. Samples with different letters in the same column are significantly different at P < 0.05*.

In contrast, the Mw and Mn of EJFSS, ECS, EPS, ERS, EWS, and EMS were significantly decreased compared to those of their raw starches (Table 2, Supplementary Table S2). Rg and PI slightly differed between the extrudate samples and raw starch samples. The different Mw and its distribution between the extrudates and native starch samples may be explained by rupture of the internal intermolecular hydrogen bonds during extrusion (19). This results in complete destruction of the amylopectin double helix backbone, leading to crystallization of semicrystalline lamella degradation of starch molecules. Hence, raw starch showed weaker resistance to digestion after extrusion (27). However, ERS and EMS showed lower Mw and Mn but higher resistance to digestion than their native starches. Based on previous reports (13, 30), the shear degradation of unconjugated side-chain branch points of amylopectin outside the starch microcrystal molecule generally resulted in decreased GI, which differed from amylopectin backbone degradation. These contrasting results for ERS and EMS may be explained by the varied degradation approaches. Liu et al. (33) found that granular molecules in rice starch are significantly degraded after extrusion cooking, which is consistent with our results.

#### Fine molecular structure of modified starch

**Figure 4:**
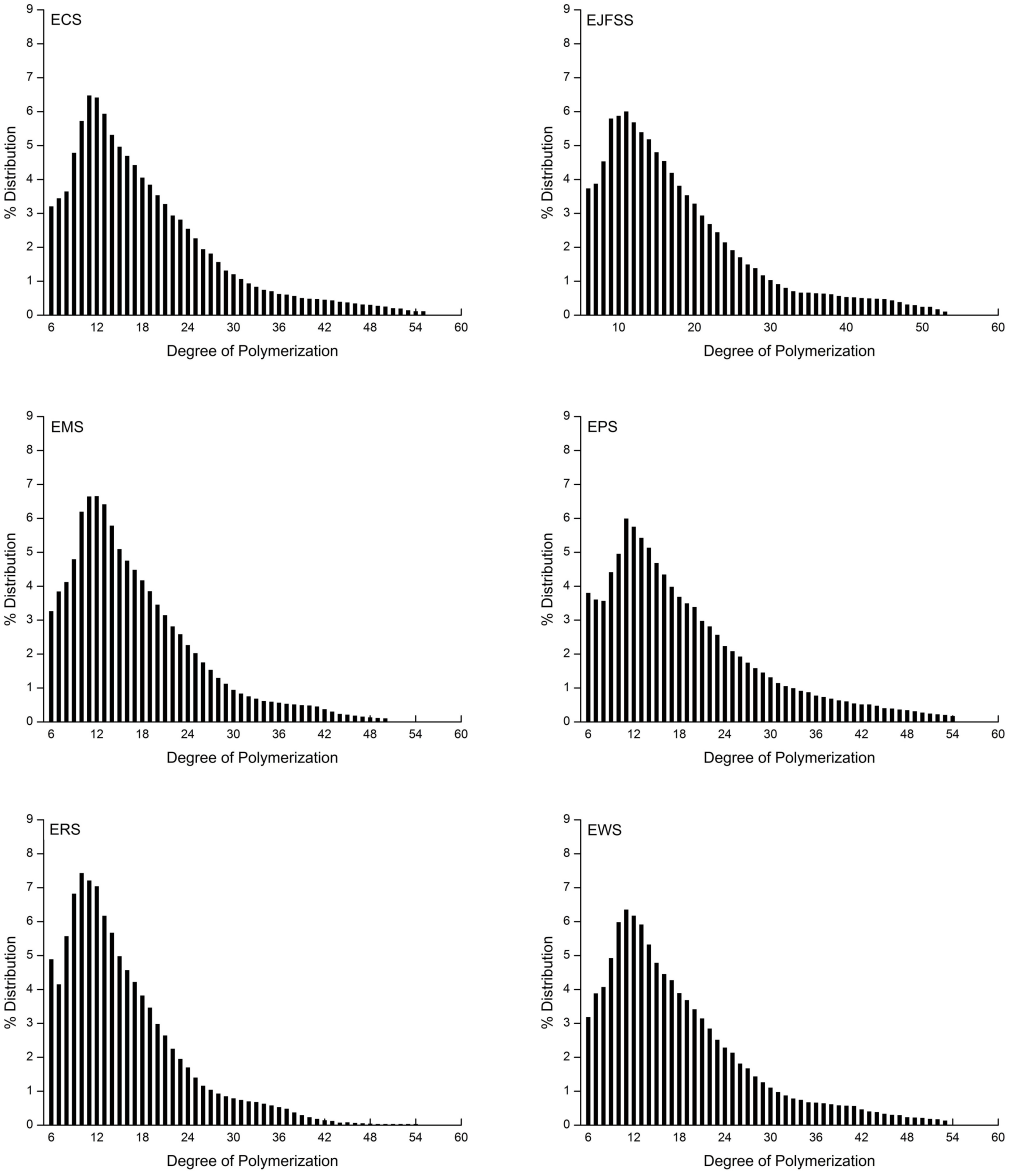
Branched chain length distributions of starch samples.

As shown in Table 2 and Figure 4, the A, B1, B2, and B3+ chain values of EJFSS, ECS, EPS, ERS, EWS, and EMS were 32.06–43.09%, 44.37–48.61%, 10–15.81%, and 2.54–7.46%, respectively. There was notable diversity between the debranched chain length distributions of the extrudate samples (*p* < 0.05). EWS, with the longest A chain lengths, had the shortest medium (B1 chains), medium-long (B2 chains), and long chains (B3+ chains) compared with that of the other extrudates. EPS showed the opposite trend, resulting in EWS having the highest GI and EPS having the lowest GI as described by Bi et al. (13). According to previous studies (2, 13), part of the medium and short chains forms a flawed crystal and amorphous layer, and both medium-long and long chains constitute a defective and perfect crystal layer in the starch semi-crystalline structure. Therefore, the different results of chain length distributions of extrudate samples resulted in diverse structures within crystallized and amorphous layers, leading to their various GI. Similarly, Lee and Moon (34) found that the A chains content in potato starch was 21.1%, proportion of B1 chains was 49.1%, and values of middle-long chains and long chains were 12.0% and 11.6% after heat-moisture treatment.

The proportion of A chains in all extrudate samples was notably increased and those of B2 and B3+ chains were notably decreased compared with those of raw samples (Table 2, Supplementary Table S1). The proportion of B1 chains in EPS, EJFSS, ECS, ERS, and EMS was higher than that in the corresponding native starches, whereas the proportion of B1 chains in EWS showed the opposite tendency. These results indicate that the B2 and B3+ chains of all native starches were transformed into A and B1 chains. For wheat starch, B1, B2, and B3+ were converted into short chains. These trends caused notable decreases in the Mw of all extrusion modified starches, which is attributed to the significant correlation between the long chains of amylopectin, Mw, and GI (35, 36). According to Zhu (35), for the Mw, fine structure, and digestibility results of raw and extrusion-cooked starches, the long helix with strong hydrogen-bonding interactions in the ordered crystal layer was clearly destroyed. This led to formation of abundant incomplete double-helix chains in the disorder phase of the semi-crystalline structure during IEMS (2, 37). However, compared to native starches, rice and maize extrudates showed a decreased order degree of crystallizing layer arrangement but an increased anti-enzymatic ability. According to Ren et al. (38), this result may be explained by the formation of abundant amylose and more stable α-1,4 glucosidic bonds units within the defective crystallizing layers of IEMS-transformed rice and maize starch. Nakazawa and Wang (37) similarly reported that the A chains value increased and values of B1, B2 and B3+ chains of maize starch decreased during extrusion cooking.

#### Crystallinity characteristic and DG analysis of starches

**Figure 5:**
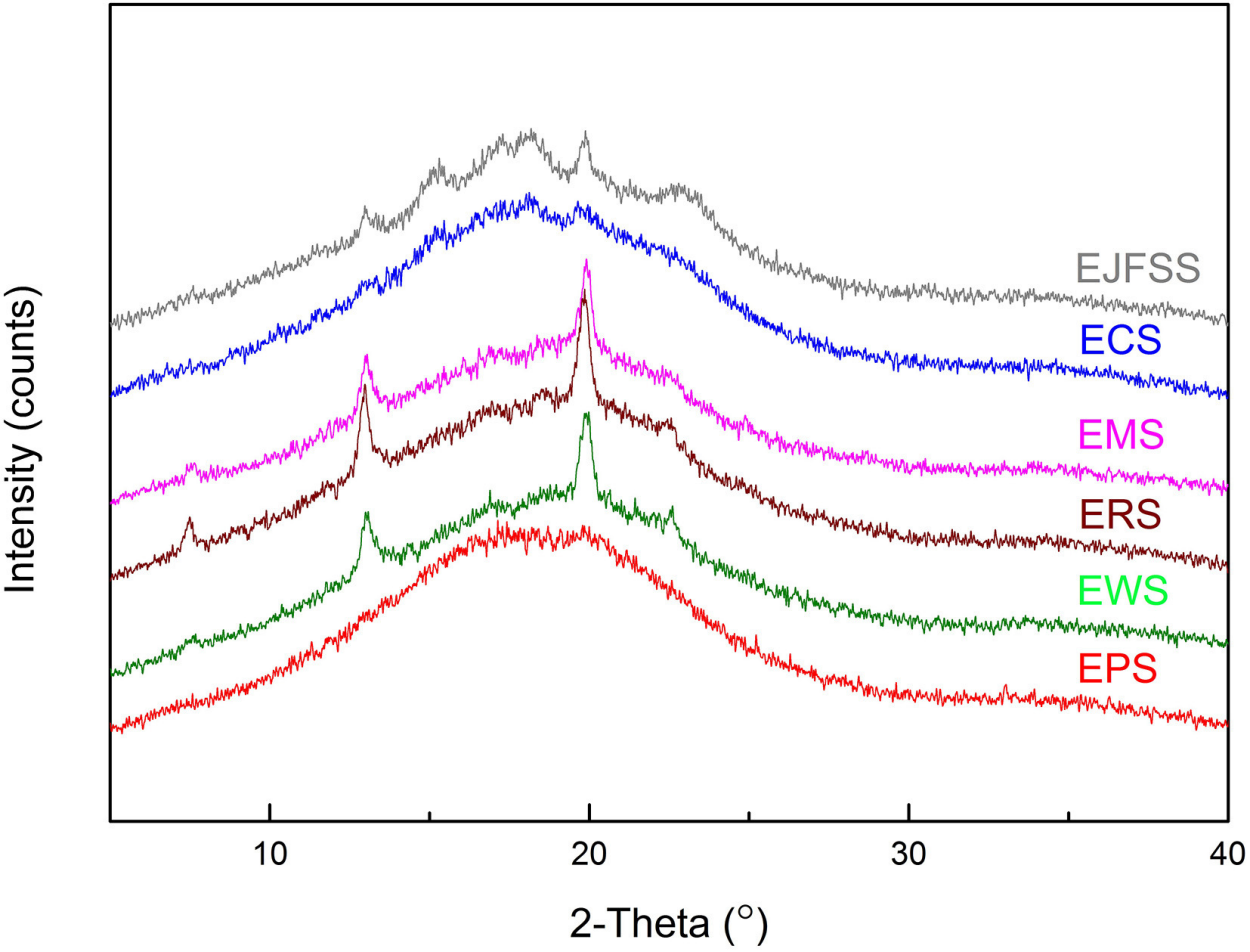
X-ray diffraction patterns of different types of starches.

As shown in Figure 5 and Table 2, Rc values were in the order EJSS (16.22%) > ECS (15.10%) > ERS (13.92%) > EWS (12.62%) > EMS (12.29%) > EPS (9.28%). According to Liu et al. (33), the differences between samples can be explained by the number of amylose lipid complex, the different degrees of close packing mode of the double-helix structure and double helix orientation within crystal lamellae, leading to various perfect crystallite numbers and sizes of each extrudate. In addition, EPS had a lower Rc but lower digestibility than that of the other starches, possibly because EPS has a larger number of superhelix structure formed by linear amylose and amylopectin compared to that of the other samples (27). A similar study reported that the Rc of extruded rice starch was 4.4–6.5% (33).

The Rc values of starch extrudates (9.28–16.22%) were notably decreased compared to those of the corresponding native samples (15.91–29.39%) (Table 2, Supplementary Table S2), indicating that the crystallization region was degraded after IEMS. The fine supramolecular structures showed that B3+ chains transformed into A, B1, and B2 chains, causing a decrease in Mw and Rc values. Therefore, molecular degradation occurred in most crystalline structures within the crystalline regions, altering the digestive rate between the raw starch and extrudates (39). Similarly, the Rc values decreased from 31.63% and 28.58% to 12.68% and 13.67% in extrusion cooked rice starch and JFSS (2, 7). Diffraction peaks of the extrudates were observed at 15, 13, 17, 18, 20, and 23° (EJFSS) or at 13 and 20° (ECS, EPS, EMS, EWS, and ERS), indicating that A- or B-type crystallinity feature changed to the V-type compared with our previous results (4). These results may be ascribed to the change in stacking modes of the open packing of helices and array of inter-helical crystal water structures in each hexagonal crystal unit (38). Similarly, Sarawong et al. (40) reported that the A-, B-, or C-type crystalline structures of green banana flour were converted into V-type structures after extrusion cooking.

In addition, the DG values of extrudates followed the order of EPS (98.84%) > EJFSS (98.05%) > ECS (97.63%) > EMS (97.11%) > ERS (96.57%) > EWS (96.08%). The different DG values of extrusion cooking samples might be the diversities of rigidity degree of native starch molecular crosslinking network formed by ordered helices and amorphous single chains. Based on Ren et al. (38), it was found that there was a small impact for DG values to digestibility of starch extrudates when DG values higher than 95%. DG values of extrusion cooking samples was higher than that of cooking foxtail millet starch (<90%) published by Ren et al. (38). This phenomenon might can be due to the high gelatinization efficiency produced by instantaneous high temperature, high pressure and high sheer force of IEMS technology, compared with traditional gelatinization technology.

### Amylose content

The proportions of amylose and its proportional relationships with amylopectin of starch followed the order EPS (31.34 and 45.65%) > EJFSS (26.98 and 36.98%) > ECS (24.08 and 31.72%) > EWS (22.50 and 29.05%) > EMS (20.56 and 25.87%) > ERS (9.63 and 10.67%) (Table 2). The amylopectin content followed the order of ERS (90.37%) > EMS (79.44%) > EWS (77.50%) > ECS (75.93%) > EJFSS (73.02%) > EPS (68.66%). The amylose content of the extrudates nearly agreed with the results for the Mw, Rs, and long chains (B2 and B3) of amylopectin. These results indicate that higher proportions of amylose, a lower value of amylopectin, and higher values of their ratios can cause higher tensile forces between adjacent amylose conformations, causing shrinkage of the amorphous regions (13, 38). Thus, the fine structures of EPS, EJFSS, and ECS were compact, indicating that these starches were digested more slowly compared to those of EWS, EMS, and ERS, which had a higher proportion of amylose. The proportions of amylose in extrusion cooked samples were similar to those previously reported for extrusion-cooked green banana starch (17.96–33.49%) (40).

The amylose contents and amylose/amylopectin ratios of ECS, EPS, and ERS were notably increased compared to those of the corresponding native samples (Table 2, Supplementary Table S3); the amylopectin contents of ECS, EPS, and ERS showed contrasting results. Sarawong et al. (40) found similar results, where the proportion of amylose in extrusion-cooked banana starch improved from 16.20 to 33.49%. The proportions of amylose and amylose/amylopectin content obtained from EJFSS, EMS, and EWS slightly differed from those of their native starches. Our results were similar to those of previous findings on the amylopectin content of amaranth flour, which changed slightly from 68.8% (native) to 69.4–69.7% (extrudates) (41). During IEMS treatment, the diverse changes in amylose and amylopectin contents between ECS, EPS, and ERS compared to the others, may be ascribed to differences in plant origins and the number of entanglements between amylose chains (19, 35). Additionally, the weight average molar mass of amylopectin accounts for more than 90% of the whole starch molecules (31). The Mw of all different types of starches was decreased during extrusion cooking, indicating that amylopectin (long chains) is degraded into amylose/amylose was sheared into amylose fragments or integral amylopectin sheared into incomplete amylopectin fragments (41). Therefore, the two different mechanisms of IEMS treatment of starch samples require further analysis.

### Mechanisms of starch samples treated using IEMS

**Figure 6:**
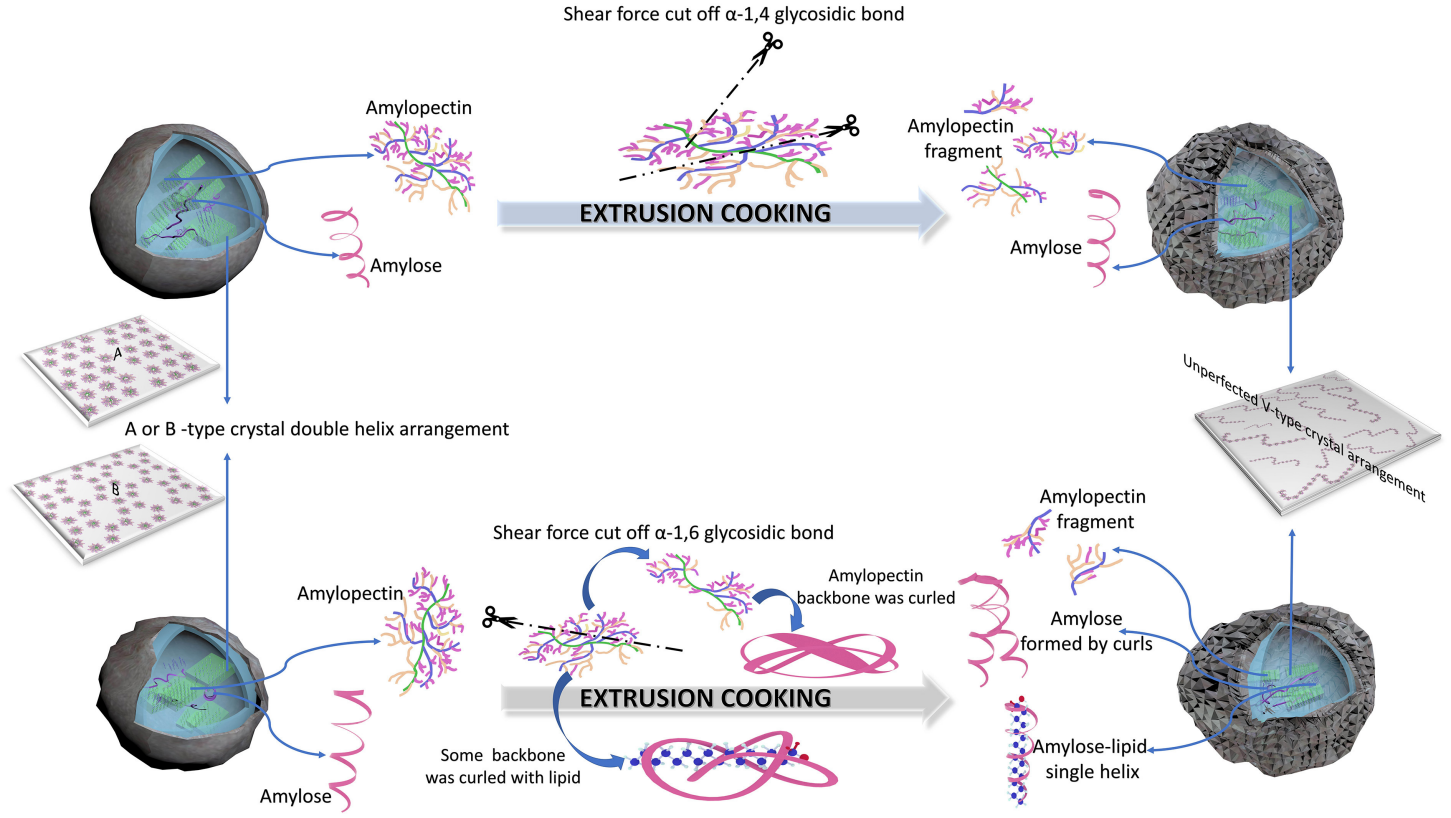
Mechanisms of improved extrusion cooking technology (IEMS) treatment of different types of starches.

Figure 6 shows the possible degradation mechanisms of IEMS treatment of different kinds of starches. For cassava, potato, and rice starches, most amylopectin α-1,6-glycosidic bonds linked to the backbone were cut off because of their increasing amylose content, decreasing the Mw and Rc in the presence of the strong shear force generated during IEMS (Figure 6, Table 2, Supplementary Table S2). According to Liu et al. (33) and Le Corre et al. (22), these residual amylopectin backbones with few short side chains may have been entangled and curled by stronger conjugation effects of non-reducing or reducing terminal glucosyl residues, converting into a spiral amylose fragment. Additionally, numerous branched chains of cleaved α-1,6-glycosidic bonds of amylopectin were converted into amylopectin fragments with a lower Mw, rather than being converted into amylose. Moreover, according to Menegassi et al. (41), few amylose-lipid complexes were generated by amylose formed from the curled amylopectin molecule, which partly slowed digestibility and stabilized few crystalline structures of ECS, EPS, and ERS. Our results agree with published results for wheat and rice starch (31, 40) but contrasted with those of jackfruit seed starch (5).

However, another degradation mechanism was observed in EJFSS, EMS, and EWS during IEMS. As shown in Figure 6, Table 2, Supplementary Table S2, the shear force produced in the extruder mainly acted on α-1,4-glycosidic bonds of long amylopectin chains by degrading them into many amylopectin fragments with lower Mw. This may be explained by their insignificant change in the amylose content, decreasing Mw, and long chains of amylopectin in all starch samples were degraded to middle and short chains during extrusion cooking (Figure 6, Table 2, Supplementary Table S2). According to Chen et al. (1) and Li et al. (42), the higher branch density of amylopectin fragments caused a stronger van der Waals force between each side chain and the corresponding backbones, preventing the amylopectin fragments from curling into amylose through a squeezing conjugation action. Therefore, amylopectin transformed into amylopectin fragments rather than amylose during IEMS. Although EMS showed a degradation pattern opposite to that of ERS, EMS also had a lower digestion rate compared to that of raw maize starch. According to Zhang et al. (3), a different superhelix formed by linear amylose and amylopectin fragments during IEMS treatment may explain this result. The degradation mechanism in EJFSS agreed with that observed previously (5).

Previous studies (2, 27, 43), found that when different types of native starches show distinct amylose values, the hydrogen-bonding forces of the amylopectin branches are significantly different owing to the dissimilar patterns in amylose and amylopectin that are self-assembled by granule-bound starch synthase I or IIa enzymes (GBSSI and SSIIa). Consequently, the higher amylose content of starch resulted in weak intermolecular hydrogen bonding within the side-chains of α-1,4-glycosidic bond of amylopectin than that of the α-1,6-glycosidic bond compared with starch of a lower amylose content (43, 44). Therefore, the different molecular degradation mechanisms during IEMT treatment between cassava, potato, and rice starches and jackfruit seed, maize, and wheat starches may be induced by various amylose contents as well as by the unraveling structural disassembly and reassembly actions of starch molecules grown from distinct plant sources. Moreover, based on the same degradation mechanism, amylopectin was cut off, and B3+ chains of amylopectin in all starch samples were degraded to B1, B2 and A chains. The declining Mw and Rc caused the broken morphology of the native starches. The mechanism of the IEMT treatment of starch samples agreed with that observed by Ji et al. (44), whose conclusion suggested that raw corn starch with a different amylose/amylopectin ratio presented a distinct mechanism of extrusion cooking.

### Interaction between glycemic release speed characteristics and fine supramolecular structure

**Figure 7:**
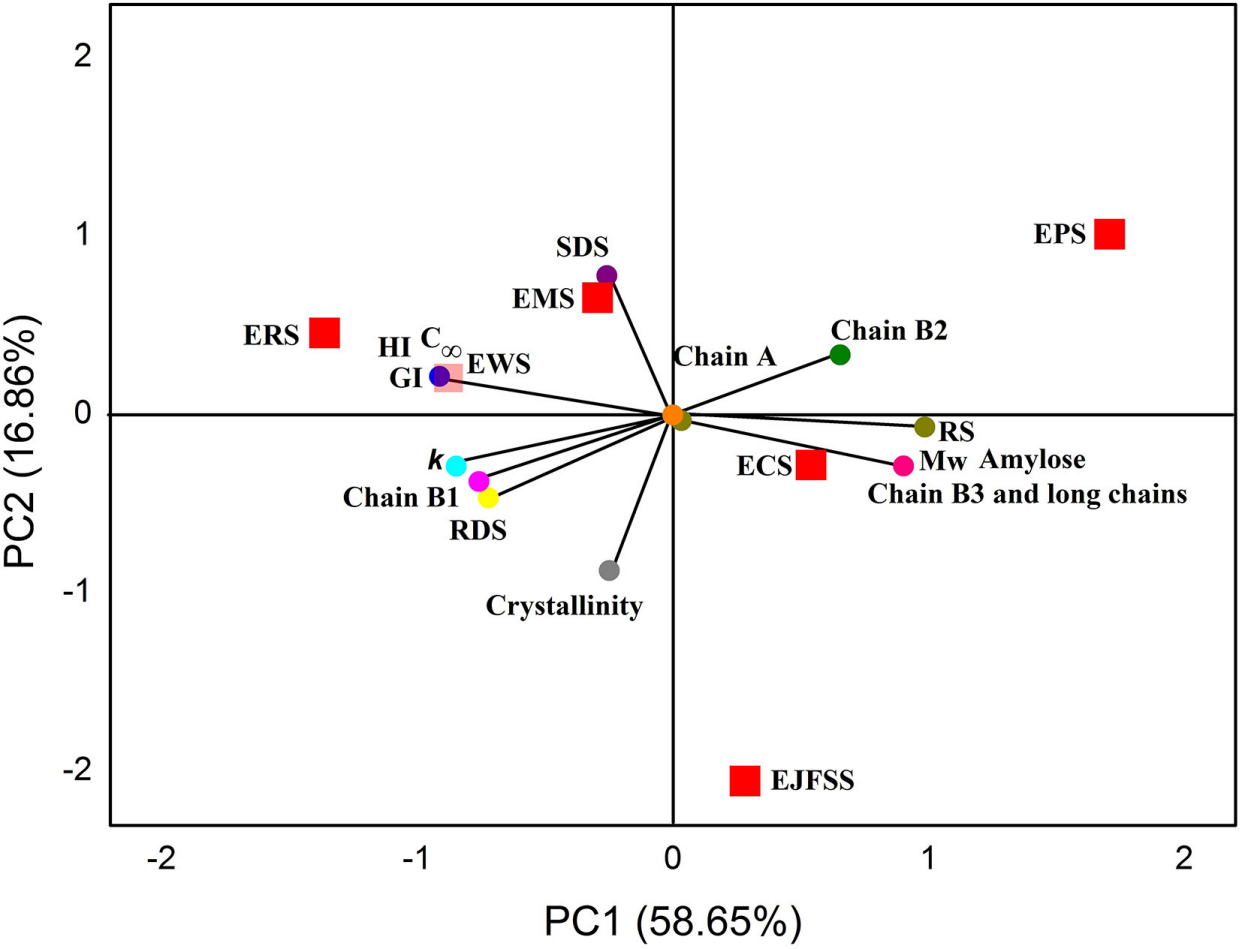
Principal component analysis (PCA) score and loading plots (PC1 and PC2) of starches.

The PCA results are shown in Figure 7, and the results were further analyzed to determine the interaction between glycemic release characteristics and fine supramolecular structure of IEMT treated samples. ERS, EMS, EWS, ECS, EJFSS, and EPS was broadly distributed in the PCA figure, indicating that differed genotypes of the native starches significantly influenced the supramolecular structures and in vitro glycemic release characteristics during IEMT treatment (13). ECS, EJFSS, and EPS were scattered in PC1, and ERS, EMS, and EWS existed in PC2. These results indicate that ECS, EJFSS, and EPS have more similar characteristics than do ERS, EMS, and EWS. Moreover, PC1 mainly included the short chain (A chain), middle-long chain (B2 chain), long chain (B3+ chain), RS, Mw, and proportion of amylose. SDS, C_∞_, HI, GI, *k*, middle-short chain, RDS, and Rc was displayed in PC2 part. A highly remarkably positive relationship was shown among the short chain, middle-long chain, long chain, RS, Mw, and proportion of amylose in PC1 (*p* < 0.05). C_∞_, HI, GI, *k*, middle-short chain, and RDS also showed a significant positive relationship (*p* < 0.05). Zabidi et al. (12) similarly reported that the C_∞_ of chempedak seed flour swelled to 15.48% from 14.19%, *k* swelled to to 0.09 *h*^−1^ to 0.07 *h*^−1^, and GI increased from 61.10 to 63.44. Short chain, middle-long chain, long chain, RS, Mw, and proportion of amylose showed a negative correlation with C_∞_, HI, GI, *k*, middle-short chain, and RDS (*p* < 0.05). SDS had a significantly negative relationship with long chain, RS, Mw, proportion of amylose, and Rc (*p* < 0.05). Lee and Moon (34) showed a similar result with present research, who found that a strong negative relationship was observed between the B3+ chain and RDS when waxy potato starch underwent heat–moisture treatment. Rc and RS had a significant negative correlation (*p* < 0.05), which agreed with a published report (13), whose investigation showed that the value RS increased from 19.04 g to 46.42 g/100 g, however, the Rc decreased to 31.41% from 41.66% for cooked banana starch. Rc had a weak correlation with the B3+ chain, Mw, and amylose content, which was similar with a previous report (33), who showed that Rc was closely associated with the molecular weight distribution of extrusion-cooked rice starch. These dissimilarities might be due to dissimilarities in the degree of stability of the chain segment conformations of starch amylopectin molecules (13). The outcomes obtained from PCA indicated that the supramolecular structure plays an important role in affecting the *in vitro* glycemic release characteristics during IEMT treatment. Furthermore, the supramolecular structures of cassava, jackfruit seed, maize, potato, rice, and wheat starches were broken, as observed from the cleavage of the α-1,4, or 1,6-glycosidic bonds of amylopectin fine structure. Accordingly, morphologies of cassava, jackfruit seed, potato, and wheat starches granules were changed from smooth and compact surfaces to looser polyhedrals with many pits and hollows; this transformation resulted in enhanced digestibility. Although the glycosidic bonds of rice and maize starch were broken and the supramolecular structures were degraded during IEMT treatment, the granule morphology had smaller and less concave holes than did that of the native starches as a result of decreased digestibility. The distinct results between ERS and EMS and the other extrudate samples can be ascribed to the lower proportion of amylose and larger pores on the surface fissures, and channels within raw rice and maize starch granules compared to those of cassava, jackfruit seed, corn, potato, and wheat starches (3, 4). Moreover, ordered chain alignment and cross-linking of ERS and EMS amylopectin might have been formed after IEMT (20, 33). Therefore, the ordered and tight cross-linking of long chains with high DP caused the formation of a rigid macromolecular network, resulting in lower *in vitro* glycemic release characteristics in extruded starch than in raw starch.

## Conclusion

The glycemic release characteristics and fine supramolecular structure for jackfruit seed, cassava, rice, wheat, potato, and maize starches modified by IEMS were evaluated. ECS, EJFSS, EPS, and EWS showed higher *in vitro* glycemic release compared to that of the corresponding native starches, whereas EMS and ERS showed the opposite trend. The A chains in the extrudates were transformed into B2 and B3+ chains, and Rc and Mw were notably decreased after extrusion cooking (*p* < 0.05). The original crystallization structure of all starch samples was altered to V-type from A-type crystallization. The amylose contents in ECS, EPS, and ERS were remarkably higher than those in corresponding native starches (*p* < 0.05), whereas changes in the amylose content of EMS, EJFSS, and EWS were minimal (*p* > 0.05). In summary, it was demonstrated that, during IEMT treatment, the α-1,4-glycosidic bonds of maize, jackfruit seed, and wheat starch amylopectin were disrupted because of the high amylose content. The lower amylose content observed for cassava, potato, and rice starches was degraded through α-1,6-glycosidic bonds. Consequently, the van der Waals forces between the branched chains were weakened, and abundant unlinear amylose and amylopectin fragments with side chains were generated. After degradation, the various reassembly patterns of amylose and amylopectin molecules resulted in improved digestibility for ECS, EJFSS, EPS, and EWS and stronger enzyme-resistance capacity for EMS and ERS. PCA further revealed the association between the supramolecular structure and *in vitro* glycemic release characteristics. Moreover, ECS, EJFSS, and EPS had a more ordered molecular structure and compact granule morphology compared to those of EWS, EMS, and ERS, resulting in lower digestibility because of the higher Mw, proportion of amylose, and long chains of amylopectin in the EWS, EMS, and ERS granules. These results may improve the utilization of starches with various GIs of different food fields for people who require different nutritional adaptations.

## Data availability statement

The original contributions presented in the study are included in the article/Supplementary material, further inquiries can be directed to the corresponding author.

## Author contributions

Conceptualization, software, and validation: CH. Formal analysis, investigation, resources, data curation, and writing-original draft preparation: BL. Writing-original draft preparation, writing-review and editing, and methodology: YZ. Visualization and supervision: WL. Project administration and funding acquisition: JL. All authors have read and agreed to the published version of the manuscript.

## Funding

This work was supported by the College of Light Industry and Food Engineering, Guangxi University, and Spice and Beverage Research Institute, Chinese Academy of Tropical Agricultural Sciences. This study was financially supported by National Key Research and Development Program (2020YFD1001204), Key Research and Development Program of Hainan Province (ZDYF2019069, ZDYF2020049), Natural Science Foundation of Guangxi Province (2019JJD120012), and Key Research and Development Plan of Guangxi (AB18221126).

## Conflict of interest

The authors declare that the research was conducted in the absence of any commercial or financial relationships that could be construed as a potential conflict of interest.

## Publisher's note

All claims expressed in this article are solely those of the authors and do not necessarily represent those of their affiliated organizations, or those of the publisher, the editors and the reviewers. Any product that may be evaluated in this article, or claim that may be made by its manufacturer, is not guaranteed or endorsed by the publisher.

## Abbreviations

ECS: extrusion cooking cassava starch
EPS: extrusion cooking potato starch
EJFSS: extrusion cooking jackfruit seed starch
EMS: extrusion cooking maize starch
EWS: extrusion cooking wheat starch
ERS: extrusion cooking rice starches
IEMS: improved extrusion cooking technology
DMSO: dimethyl sulfoxide
HI: hydrolysis index
GI: glycemic index
RDS rapidly digestible starch
SDS slowly digestible starch
RS: resistant starch
SDI: starch digestible index
C_∞_: equilibrium concentration
*k*: enzymatic hydrolysis speed rate
Mw: weight-average molar mass
Mn: number-average molar mass
Rg: radius of gyration
Rc: relative crystallinity
PCA: principal component analysis.
