# Supplementary material for: Effect of new type extrusion modification technology on supramolecular structure and in vitro glycemic release characteristics of starches with various estimated glycemic indices
Source: Front Nutr. 2022 Aug 15;9:985929. doi: 10.3389/fnut.2022.985929 (PMC9423736; doi:10.3389/fnut.2022.985929)
Supplement: Supplementary file 1 [file Data_Sheet_1.pdf]

Supplemental tables

Table S1 In vitro kinetics of starch hydrolysis

Time	EJFSS (%)	ECS (%)	EPS (%)	EMS (%)	EWS (%)	ERS (%)
0	0	0	0	0	0	0
10	26.52±0.10^{e}	22.44±0.11^{g}	19.23±25.36^{f}	25.36±5.55^{g}	30.84±1.18^{g}	30.99±2.57^{g}
20	35.69±0.15^{d}	32.83±1.74^{f}	28.00±36.56^{e}	36.56±5.18^{f}	42.92±0.59^{f}	46.63±1.05^{f}
30	49.75±0.25^{c}	54.53±1.12^{e}	42.66±50.51^{d}	50.50±3.77^{e}	56.22±2.59^{e}	55.96±0.69^{e}
60	70.59±2.12^{b}	62.57±0.93^{d}	59.21±68.37^{c}	68.37±4.03^{d}	74.25±0.98^{d}	78.80±0.78^{cd}
90	77.81±3.28^{a}	75.35±0.27^{c}	69.74±78.29^{b}	78.29±1.20^{c}	87.65±0.95^{bc}	82.27±0.67^{c}
120	79.09±1.93^{a}	80.63±1.13^{b}	72.49±85.36^{b}	85.36±3.49^{b}	89.95±1.26^{ab}	87.44±0.49^{b}
180	83.38±1.38^{a}	88.95±1.83^{a}	80.33±93.61^{a}	93.61±2.44^{a}	93.95±1.41^{a}	96.39±0.77^{a}

Samples with different letters in the same column are significantly different at $P < 0.05$.

Table S2 In vitro nutritionally starch fractions and model parameters of part of native different kinds of starch, calculated equilibrium concentration (C_∞), enzymatic hydrolysis speed rate (k), hydrolysis index (HI) and glycaemic index (GI) (1).

Samples	C_∞ (%)	k (h^{-1})	HI	GI	RDS (%)	SDS (%)	RS (%)
JFSS	35.80±1.44^{e}	0.62±0.10^{c}	41.43±3.36^{e}	62.45±1.84^{e}	3.93±0.29^{e}	18.47±0.40^{e}	77.60±0.12^{b}
CS	49.23±1.16^{d}	0.50±0.02^{e}	57.22±1.36^{d}	71.13±0.15^{d}	9.07±1.46^{d}	38.94±2.92^{d}	51.99±4.66^{c}
PS	7.47±0.34^{f}	0.48±0.04^{f}	8.67±0.40^{f}	44.48±0.22^{f}	1.40±0.01^{f}	3.86±0.23^{f}	94.74±0.20^{a}
MS	92.05±2.30^{a}	0.58±0.06^{d}	107.11±4.25^{a}	98.52±3.43^{a}	17.94±2.07^{c}	58.09±0.58^{a}	23.97±5.67^{d}
WS	82.29±3.86^{c}	0.70±0.07^{b}	95.87±4.55^{c}	92.34±2.50^{c}	24.20±1.22^{b}	55.49±2.78^{c}	20.10±1.66^{e}
RS	91.06±2.67^{b}	1.02±0.07^{a}	106.08±2.94^{ab}	97.95±1.61^{ab}	37.27±1.01^{a}	58.06±1.64^{ab}	4.66±2.83^{f}

JFSS, jackfruit seed starches; CS, cassava starch; PS, potato starch; MS, maize starch; WS, wheat starch; RS rice starch. RDS, rapidly digestible starch, SDS, slowly digestible starch, RS, resistant starch, SDI, starch digestible index. Samples means with different letters in the same column are significantly different at $P < 0.05$.

Table S3 The content of amylose, amylopectin, their ratio, relative crystallinity (RC), ranched chain length distributions and molecular weight of native different starch (1).

Starch sample	Amylose (%)	Amylopectin (%)	Ratio (%)	RC (%)	DP 6-12	DP 13-24	DP 25-36	DP ≧ 37	Mn ($\times 10^7$)	Mw ($\times 10^7$)	Rg (nm)	PI
JFSS	27.01±0.67[a]	72.99±0.67[e]	37.00±1.26[a]	29.39[a]	32.75[a]	36.08[f]	18.32[f]	12.83[ab]	1.85[b]	2.09[b]	115.7[a]	1.13[d]
CS	16.47±0.85[d]	83.53±0.85[b]	19.72±1.22[d]	28.33[b]	20.73[c]	42.63[e]	23.96[a]	12.68[bc]	0.42[e]	1.52[c]	93.0[b]	3.60[a]
PS	24.82±1.15[b]	75.19±1.15[d]	33.02±2.04[b]	15.91[f]	19.20[de]	44.54[d]	22.71[bc]	13.55[a]	3.48[a]	4.74[a]	116.4[a]	1.36[c]
MS	21.13±0.99[c]	78.87±0.99[c]	26.80±1.59[c]	25.19[d]	19.33[d]	47.96[bc]	21.79[cd]	10.92[d]	0.63[c]	1.20[d]	86.1[c]	1.90[b]
WS	21.65±0.40[c]	78.35±0.40[c]	27.63±0.65[c]	22.95[e]	16.56[f]	50.02[a]	23.02[ab]	10.40[de]	0.58[cd]	1.06[d]	65.8[e]	1.82[b]
RS	3.25±0.06[e]	96.75±1.06[a]	3.36±0.09[e]	26.49[c]	27.41[b]	46.41[b]	20.02[e]	6.17[f]	0.33[f]	0.42[e]	75.5[d]	1.26[cd]

Ratio, Amylose/Amylopectin. Samples means with different letters in the same column are significantly different at $P < 0.05$.

1. Li B, Wang H, Wang X, Zhang Y, Tan Y, Zhang Y, Chu Z, Zhang Y. Prediction of the Postprandial Blood Sugar Response Estimated by Enzymatic Kinetics of In Vitro Digestive and Fine Molecular Structure of Artocarpus heterophyllus Lam Seed Starch and Several Staple Crop Starches. *Starch/Staerke* (2019) 71: doi: 10.1002/star.201800351
